# Mesenchymal stem cells enhance CCL8 expression by podocytes in lupus-prone MRL.*Fas*^lpr^ mice

**DOI:** 10.1038/s41598-023-40346-8

**Published:** 2023-08-11

**Authors:** Hyung Sook Kim, Hong Kyung Lee, Kihyeon Kim, Gi Beom Ahn, Min Sung Kim, Tae Yong Lee, Dong Ju Son, Youngsoo Kim, Jin Tae Hong, Sang-Bae Han

**Affiliations:** 1 College of Pharmacy, Chungbuk National University, Cheongju, Chungbuk 28160 Republic of Korea; 2 Department of Biotechnology and Biomedicine, Chungbuk Provincial University, Cheongju, Chungbuk 28160 Republic of Korea; 3 Bioengineering Institute, Corestem Inc., Gyeonggi, 13486 Republic of Korea

**Keywords:** Immunology, Stem cells, Nephrology

## Abstract

Nephritis is common in systemic lupus erythematosus patients and is associated with hyper-activation of immune and renal cells. Although mesenchymal stem cells (MSCs) ameliorate nephritis by inhibiting T and B cells, whether MSCs directly affect renal cells is unclear. To address this issue, we examined the direct effect of MSCs on renal cells with a focus on chemokines. We found that expression of CCL2, CCL3, CCL4, CCL5, CCL8, CCL19, and CXCL10 increased 1.6–5.6-fold in the kidney of lupus-prone MRL.*Fas*^lpr^ mice with advancing age from 9 to 16 weeks. Although MSCs inhibited the increase in the expression of most chemokines by 52–95%, they further increased CCL8 expression by 290%. Using renal cells, we next investigated how MSCs enhanced CCL8 expression. CCL8 was expressed by podocytes, but not by tubular cells. MSCs enhanced CCL8 expression by podocytes in a contact-dependent manner, which was proved by transwell assay and blocking with anti-VCAM-1 antibody. Finally, we showed that CCL8 itself activated MSCs to produce more immunosuppressive factors (IL-10, IDO, TGF-β1, and iNOS) and to inhibit more strongly IFN-γ production by T cells. Taken together, our data demonstrate that MSCs activate podocytes to produce CCL8 in a contact-dependent manner and conversely, podocyte-derived CCL8 might potentiate immunosuppressive activity of MSCs in a paracrine fashion. Our study documents a previously unrecognized therapeutic mechanism of MSCs in nephritis.

## Introduction

Systemic lupus erythematosus (SLE) is a multi-organ autoimmune disease characterized by autoantibody production to ubiquitous self-antigens^1^. Nephritis occurs in more than 50% of SLE patients and is associated with increased morbidity and mortality^2^. Nephritis is initiated by the deposition of immune (antigen–antibody) complexes in the kidney, which activates renal cells, such as tubular cells and podocytes, to produce inflammatory chemokines^3^. Chemokines are considered as key molecules that can amplify renal inflammation and damage^4,5^. CCL2, CCL3, CCL5, CXCL10, and CXCL16 are increased in lupus nephritis patients and lupus-prone MRL.*Fas*^lpr^ mice^6,7^. Podocytes produce CCL2, CCL7, CXCL1, and CXCL5, which then recruit inflammatory immune cells, such as T cells, B cells, natural killer (NK) cells, neutrophils, monocytes, and dendritic cells, to the kidney^8^. Inflammatory cytokine TNF-α is a common trigger for the production of chemokines by renal cells^9^.

An important aspect of mesenchymal stem cell (MSC) therapy relevant to SLE is the effects of these cells on immune cell functions. MSCs inhibit the proliferation of and cytokine production by T cells, the proliferation of and antibody secretion by B cells, dendritic cell maturation, and NK cell functions^10^. MSCs inhibit T cell functions by producing immunosuppressive factors and by direct cell–cell contacts. The soluble mediators produced by MSCs are TGF-β, prostaglandin E_2_ (PGE_2_), indoleamine 2,3-dioxygenase (IDO), IL-10, and nitric oxide, all of which inhibit the activation of inflammatory leukocytes^11^. MSCs also increase the proliferation and functions of Tregs. Overall, these properties of MSCs make them good candidates for the treatment of SLE^11^.

In our previous study, we showed that MSCs ameliorate nephritis in MRL.*Fas*^lpr^ mice and demonstrated that the underlying mechanism is the inhibition of T cell functions by MSCs in a CCL2-dependent manner^12^. Although naïve MSCs do not inhibit B cells, MSCs primed with IFN-γ or phorbol ester inhibit B cells in a CXCL10- and PD-L1-dependent manner^13,14^. Although the inhibitory effects of MSCs on T and B cells have been extensively studied, it is still unclear whether MSCs directly affect the functions of renal cells. Here, we addressed this issue by focusing on the effects of MSCs on chemokine expression by podocytes and found a previously unreported mechanism though which MSCs ameliorated nephritis.

## Results

### Therapeutic effects of MSCs in MRL.***Fas***^lpr^ mice

**Figure 1:**
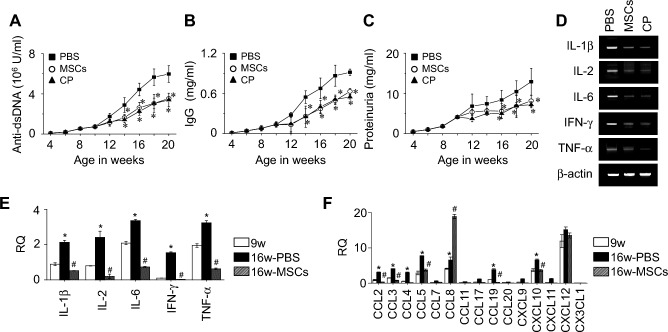
Effect of MSCs on chemokine expression profiles in the kidney of MRL.*Fas*^lpr^ mice. (**A**–**D**) Mice were intravenously injected with PBS, MSCs (1 × 10^6^ cells/mouse), or cyclophosphamide (CP, 50 mg/kg) three times at 3-week intervals from 12 weeks of age and sacrificed at the age of 20 weeks (n = 6). The serum levels of (**A**) anti-dsDNA antibody and (**B**) total IgG antibody and (**C**) urine protein levels were measured. (**D**) Total RNA was isolated from the spleen cells and the expression of inflammatory cytokines was examined by RT-PCR. (**E**, **F**) Mice were intravenously injected with PBS or MSCs (n = 6) at the age of 12 and 14 weeks, and the kidney was isolated at the age of 16 weeks (16w-PBS or 16w-MSCs). Kidney was also isolated from control mice at the age of 9 weeks (9w). Total RNA was isolated, and the expression levels of (**E**) cytokines and (**F**) chemokines were determined by RT-qPCR. RQ, Relative quantitation. (**A**–**D**) *p < 0.01: versus PBS-treated mice. (**E**, **F**) *p < 0.01 versus 9w and ^#^*p* < 0.01 versus 16w-PBS.

In our previous study, we showed that human MSCs prolonged survival, decreased the serum levels of anti-dsDNA and total IgG antibodies, and ameliorated nephritis in lupus-prone MRL.*Fas*^lpr^ mice^12^. First, we confirmed the therapeutic activity of MSCs in these mice. The serum levels of anti-dsDNA (Fig. 1A) and total IgG antibodies (Fig. 1B) and the amount of protein in the urine (Fig. 1C) were all lower in MSC-treated mice than in PBS-treated control mice. At 20 weeks of age, expression of all inflammatory cytokines tested (IL-1β, IL-2, IL-6, IFN-γ, and TNF-α) was lower in the spleen cells of MSC-treated mice when than in those of PBS-treated mice (Fig. 1D). Cyclophosphamide showed a therapeutic effect similar to that of MSCs (Fig. 1A–D). Overall, these data demonstrate that MSCs ameliorate lupus symptoms in MRL.*Fas*^lpr^ mice.

### Expression profile of chemokines in the kidney of MSC-treated MRL.***Fas***^lpr^ mice

Next, we injected MSCs into the mice at the ages of 12 and 14 weeks and isolated the kidney at the age of 16 weeks. For comparison, we also isolated kidneys from 9-week-old mice. The expression levels of inflammatory cytokines (IL-1β, IL-2, IL-6, IFN-γ, and TNF-α) in the kidney were low at 9 weeks and increased by 16 weeks of age (Fig. 1E). This increase was inhibited by MSC injection (Fig. 1E). We also found that expression of CCL2, CCL3, CCL4, CCL5, CCL8, CCL19, and CXCL10 increased 1.6–5.6-fold in the kidney of lupus-prone MRL.*Fas*^lpr^ mice with advancing age from 9 to 16 weeks (Fig. 1F). Although MSCs inhibited the increase in the expression of most chemokines by 52–95%, they further increased CCL8 expression by 290% (Fig. 1F). Overall, these data demonstrate that MSCs ameliorate lupus symptoms in MRL.*Fas*^lpr^ mice by inhibiting the expression of inflammatory cytokines and chemokines in the kidney, with the exception of CCL8.

### CCL8 is expressed by podocytes but not by tubular cells

**Figure 2:**
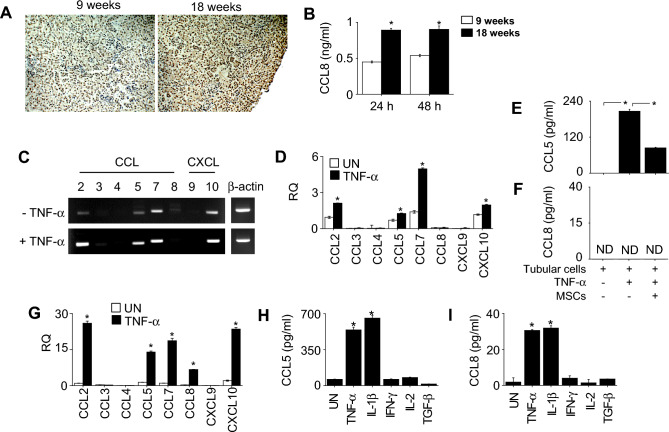
Chemokine expression by tubular cells and podocytes isolated from the kidney of MSC-treated MRL.*Fas*^lpr^ mice. (**A**, **B**) Kidneys were isolated from 9- or 18-week-old mice. (**A**) Kidney sections were stained with primary antibody against CCL8. (**B**) Small pieces of kidney tissue (100 mg) were incubated for 24 or 48 h and the levels of CCL8 in the medium were determined by ELISA. (**C**–**F**) Tubular cells were isolated at the age of 12 weeks and treated with TNF-α for 4 h. Total RNA was isolated and the expression levels of chemokines were determined by (**C**) RT-PCR and (**D**) RT-qPCR. Tubular cells (1 × 10^5^ cells/well) and MSCs (0.1 × 10^5^ cells/well) were co-cultured in the presence or absence of TNF-α for 24 h and the levels of (**E**) CCL5 and (**F**) CCL8 in the medium was determined by ELISA. (**G**–**I**) Podocytes were isolated at the age of 12 weeks and treated with TNF-α for 4 h. (**G**) Total RNA was isolated and the expression levels of chemokines were determined by RT-qPCR. Podocytes were activated with the indicated cytokines (all at 50 ng/ml) for 24 h, and the levels of (**H**) CCL5 and (**I**) CCL8 in the medium were determined by ELISA. TNF-α was used at 50 ng/ml in all experiments. n = 3 in all panels. RQ, relative quantitation. UN, untreated. ND, not detectable. *p < 0.01: (**B**) versus 9 weeks; (**E**) as indicated; (**D**, **G**, **H**, and **I**) versus UN.

Next, we focused on the role of CCL8 in the nephritic kidney. Immunohistochemical analysis demonstrated that CCL8 expression in the kidney was higher at 18 weeks than at 9 weeks of age (Fig. 2A). Tissue culture experiments confirmed that kidneys from 18-week-old mice produced more CCL8 than kidneys from 9-week-old mice (Fig. 2B). Then, we isolated tubular cells and podocytes from the kidney of control MRL.*Fas*^lpr^ mice at the age of 12 weeks and examined which cell type expressed CCL8. The expression levels of CCL2, CCL5, CCL7, and CXCL10 were increased by TNF-α treatment in tubular cells in RT-PCR (Fig. 2C) and RT-qPCR (Fig. 2D). ELISA revealed that MSCs inhibited the production of CCL5 (Fig. 2E) but did not affect that of CCL8 (Fig. 2F) by tubular cells. The expression levels of CCL2, CCL5, CCL7, CCL8, and CXCL10 in podocytes were also increased by TNF-α (Fig. 2G); thus, unlike in tubular cells, CCL8 expression in podocytes was increased by TNF-α treatment (Fig. 2G). IL-1β also activated production of CCL5 (Fig. 2H) and CCL8 (Fig. 2I) by podocytes. Overall, these data suggest that, although diverse chemokines are expressed by tubular cells and podocytes, CCL8 is expressed by podocytes but not by tubular cells.

### MSCs enhance the expression of CCL8 by podocytes in a contact-dependent manner

**Figure 3:**
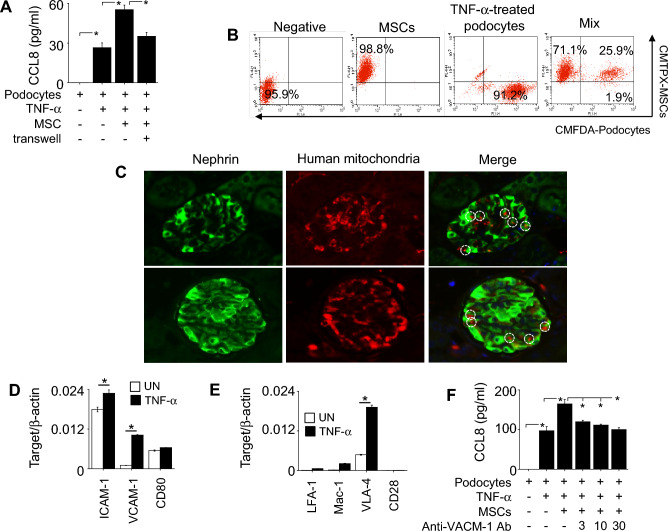
Effect of MSCs on CCL8 production by podocytes. (**A**) Podocytes (1 × 10^5^ cells/well) were loaded into the lower wells of transwell plates. MSCs (0.1 × 10^5^ cells/well) were added to the upper wells to prevent contact or to the lower wells to allow contact with podocytes. After incubation for 24 h, CCL8 level in the medium was determined by ELISA. (**B**) Binding rates of CMTPX-labeled MSCs (1 × 10^6^ cells/tube) and CMFDA-labeled podocytes (1 × 10^6^ cells/tube) were analyzed by flow cytometry. Representative dot plots are shown. (**C**) Mice were intravenously injected with human MSCs (1 × 10^6^ cells/mouse) at the age of 12 weeks (n = 6). Kidneys were isolated 24 h later. Kidney sections were stained with rabbit anti-mouse nephrin antibody conjugated with GFP (green) and mouse anti-human mitochondria antibody conjugated with Alexa Fluor 647 (red). Representative contacts are shown in circles. (**D**) The levels of ICAM-1, VCAM-1, and CD80 mRNAs in TNF-α-treated podocytes were determined by RT-qPCR. (**E**) The levels of LFA-1, Mac-1, VLA-4, and CD28 mRNAs in TNF-α-treated MSCs were determined by RT-qPCR. (**F**) Podocytes were pre-treated with anti-VCAM-1 antibody at 3–30 ng/ml for 2 h. After washing, podocytes (1 × 10^5^ cells/well) were co-cultured with MSCs (0.1 × 10^5^ cells/well). After incubation for 24 h, CCL8 level in the medium was determined by ELISA. TNF-α was used at 50 ng/ml in all experiments. RQ, Relative quantitation. UN, Untreated. n = 3 in panels of (**A**–**B**) and (**D**–**F**). *p < 0.01.

Next, we examined how MSCs enhanced CCL8 production by podocytes. When we allowed cell–cell contacts by loading both types of cells in the same wells, MSCs enhanced CCL8 production by TNF-α-treated podocytes (Fig. 3A). However, when we prevented cell–cell contacts using transwell plates, MSCs had no effect (Fig. 3A), implying a contact-dependent mechanism. Our in vitro binding assay confirmed the cell–cell contacts between MSCs and TNF-α-treated podocytes (Fig. 3B). To verify that these contacts occur in vivo, we injected human MSCs into MRL.*Fas*^lpr^ mice and showed that human mitochondria–positive MSCs (red) co-localized with nephrin-positive podocytes (green) in the kidney, suggesting contact between these cells (Fig. 3C). Since cell–cell interactions often depend on the expression of adhesion molecules^15^, we examined the expression of several adhesion molecules. TNF-α-treated podocytes highly expressed ICAM-1 and VCAM-1 (Fig. 3D). TNF-α-treated MSCs highly expressed VLA-4, a ligand of VCAM-1 (Fig. 3E) and anti-VCAM-1 antibody abolished the ability of MSCs to enhance CCL8 production by podocytes (Fig. 3F). Overall, these data suggest that MSCs activate podocytes to produce CCL8 in a VLA4-dependent manner.

### CCL8 potentiates the immunosuppressive activity of MSCs

**Figure 4:**
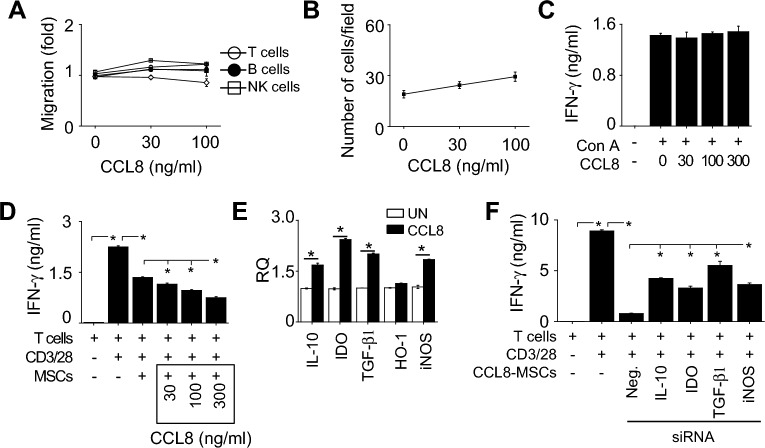
Effect of CCL8 on immune cells and MSCs. (**A**) T cells, B cells, and NK cells (1 × 10^5^ cells/well) were added to the upper wells of transwell plates. CCL8 was added to the lower wells at 30 or 100 ng/ml. The number of cells that migrated to the lower wells over 2 h was counted using a flow cytometer. (**B**) MSCs (2 × 10^4^ cells/well) were added to the upper wells of transwell plates. CCL8 was added to the lower wells at 30 or 100 ng/ml. The number of migrating MSCs was counted as described in the “Materials and methods”. (**C**) T cells isolated from the spleen of MRL.*Fas*^lpr^ mice were activated with concanavalin A (Con A, 1 μg/ml) in the presence or absence of recombinant CCL8 for 72 h. IFN-γ level in the medium was determined by ELISA. (**D**) MSCs were pretreated with recombinant CCL8 at 30–300 ng/ml for 24 h. T cells (1 × 10^5^ cells/well) were co-cultured with MSCs (1 × 10^4^ cells/well) in the presence of anti-CD3 and anti-CD28 antibodies (1 μg/ml each) for 72 h. IFN-γ level in the medium was determined by ELISA. (**E**) MSCs were treated with recombinant CCL8 (300 ng/ml). The levels of mRNAs of the indicated soluble factors were determined by RT-qPCR. (**F**) MSCs were pretreated with recombinant CCL8 (300 ng/ml; CCL8-MSCs). CCL8-MSCs were transfected with negative-control (neg), IL-10, IDO, TGF-β1, or iNOS siRNA. T cells (1 × 10^5^ cells/well) were co-cultured with MSCs (0.1 × 10^5^ cells/well) for 72 h. Anti-CD3 and anti-CD28 antibodies (1 μg/ml each) were added to activate T cells. IFN-γ level in the medium was determined by ELISA. RQ, Relative quantitation. UN, Untreated. n = 3 in all panels. *p < 0.01.

Then, we examined the effect of CCL8 on immune cells and MSCs. Recombinant CCL8 did not affect the migration of T cells, B cells, NK cells (Fig. 4A), or MSCs (Fig. 4B). Recombinant CCL8 did not affect IFN-γ production by concanavalin A (Con A)-treated T cells (Fig. 4C). However, CCL8-pretreatment enhanced the ability of MSCs to inhibit IFN-γ production by CD3/CD28-treated T cells in a dose-dependent manner (Fig. 4D). Recombinant CCL8 directly activated MSCs to highly express the immunosuppressive factors IL-10, IDO, TGF-β1, and iNOS (Fig. 4E). Transfection of CCL8-treated MSCs with siRNAs against these factors weakened their inhibitory effect on T cells (Fig. 4F). Overall, these data suggest that CCL8 activates MSCs to enhance their production of immunosuppressive factors.

### MSCs inhibit the expression of CCL5 by podocytes in a soluble factor–dependent manner

**Figure 5:**
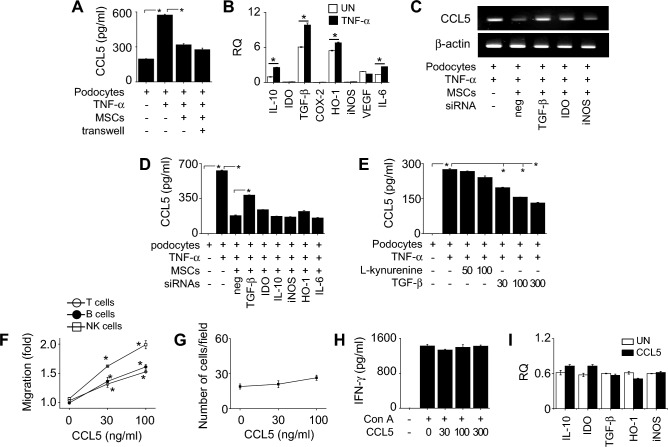
Effect of CCL5 on immune cells and MSCs. (**A**) Podocytes (1 × 10^5^ cells/well) were loaded into the lower wells of transwell plates. MSCs (1 × 10^4^ cells/well) were added to the upper wells to prevent contact or to the lower wells to allow contact with podocytes. After incubation for 24 h, CCL5 level in the medium was determined by ELISA. (**B**) The levels of mRNAs of the indicated soluble factors in MSCs were determined by RT-qPCR. (C, D) MSCs were transfected with negative-control, TGF-β, IDO, or iNOS siRNAs. MSCs (1 × 10^4^ cells/well) were co-cultured with podocytes (1 × 10^5^ cells/well). After incubation for 24 h, (**C**) expression of CCL5 by podocytes was determined by RT-PCR and (**D**) CCL5 level in the medium was determined by ELISA. (**E**) Podocytes (1 × 10^5^ cells/well) were treated with L-kynurenine or recombinant TGF-β1. After incubation for 24 h, CCL5 level in the medium was determined by ELISA. (**F**) T cells, B cells, or NK cells (1 × 10^5^ cells/well) were added to the upper wells of transwell plates. Recombinant CCL5 was added to the lower wells at 30 or 100 ng/ml. The number of immune cells that had migrated to the lower wells over 2 h was counted using a flow cytometer. (**G**) MSCs (2 × 10^4^ cells/well) were added to the upper wells of transwell plates. Recombinant CCL5 was added to the lower wells at 30 or 100 ng/ml. The number of migrating MSCs was counted as described in the “Materials and methods”. (**H**) T cells isolated from the spleen of MRL.*Fas*^lpr^ mice were activated with concanavalin A (Con A, 1 μg/ml) in the presence or absence of recombinant CCL5 for 72 h. IFN-γlevel in the medium was determined by ELISA. (**I**) Recombinant CCL5 was added to the culture of MSCs for 24 h. The levels of mRNAs of the indicated soluble factors were determined by RT-qPCR. TNF-α was used at 50 ng/ml in all experiments. RQ, Relative quantitation. UN, Untreated. n = 3 in all panels. *p < 0.01.

When we prevented the direct contact between MSCs and TNF-α-treated podocytes by using transwell plates, MSCs still inhibited CCL5 production by TNF-α-treated podocytes (Fig. 5A), suggesting a soluble factor–dependent inhibition mechanism. TNF-α increased the expression of immunosuppressive soluble factors IL-10, TGF-β1, HO-1, and IL-6 by MSCs, although these factors were expressed by MSCs in the absence of TNF-α (Fig. 5B). To assess the role of these soluble factors, we used siRNAs to knockdown their expression in MSCs. MSCs transfected with TGF-β1 siRNA only weakly inhibited CCL5 production by podocytes, whereas the other siRNAs had no significant effect (Fig. 5C and D), implying the key role of TGF-β1. Recombinant TGF-β1 inhibited CCL5 production by TNF-α-treated podocytes in a concentration-dependent manner, but kynurenine (IDO product) did not (Fig. 5E). Next, we examined the direct effect of CCL5 on immune cells and MSCs. Recombinant CCL5 increased the migration of T cells, B cells, and NK cells (Fig. 5F), but not MSC migration (Fig. 5G). Recombinant CCL5 did not affect IFN-γ production by Con A-treated T cells (Fig. 5H) or expression of immunosuppressive factors (IL-10, IDO, TGF-β1, HO-1, and iNOS) by MSCs (Fig. 5I). Overall, these data suggest that MSCs inhibit CCL5 expression by podocytes in a TGF-β1-dependent manner, which might prevent the infiltration of inflammatory immune cells into the nephritic kidney.

## Discussion

The results of this study provide several insights into the therapeutic mechanisms of MSCs in lupus nephritis. First, we verified that MSCs ameliorated nephritis by inhibiting the expression of many inflammatory cytokines in the kidney of MRL.*Fas*^lpr^ mice. We also documented two previously unrecognized mechanisms with a focus on chemokines: (1) MSCs directly inhibited CCL5 expression by podocytes in a TGF-β1-dependent manner and (2) MSCs enhanced CCL8 expression by podocytes in a VLA-4-dependent manner. We also demonstrated that CCL8 activated MSCs to enhance their production of immunosuppressive factors.

In this study, we focused on investigating how MSCs regulate chemokine expression in the nephritic kidney. Chemokines are a group of low-molecular-weight cytokines whose main function is the regulation of leukocyte migration^16^. Under healthy conditions, chemokines regulate mainly the migration of naïve immune cells to lymphoid organs and to certain peripheral tissues for normal immune surveillance^16^. In the presence of inflammation, activated immune cells in the blood up-regulate chemokine receptors and migrate into the inflamed tissues by recognizing the corresponding chemokines secreted from these inflamed tissues^16^. For example, secretion levels of CCL2, CCL3, CCL4, CCL5, CCL19, CXCL10, and CXCL12 are increased in the kidney of lupus-prone mice and SLE patients during the development of nephritis^17–22^. In lupus-prone NZB/W F1 mice, T cells and monocytes expressing CCR1 and CCR5 migrate to the kidney via sensing CCL3, CCL4, and CCL5^20^. In MRL.*Fas*^lpr^ mice, a CCR1 antagonist reduces nephritis by decreasing infiltration of T cells and monocytes^21^. CXCR4-expressing B and T cells migrate from blood to the nephritic kidney of NZB/W F1 mice, and administration of anti-CXCL12 antibodies in these mice reduces nephritis^22^. The expression levels of CCR2 and CXCR3 on leukocytes are increased in the kidney of SLE patients and lupus-prone mice^18^. Consistently, our in vivo data showed that the expression levels of CCL2, CCL3, CCL4, CCL5, CCL19, and CXCL10 increased in the kidney of MRL.*Fas*^lpr^ mice during the development of nephritis. We also demonstrated that MSCs ameliorated nephritis in MRL.*Fas*^lpr^ mice by inhibiting the expression of these inflammatory chemokines. A striking and previously unrecognized observation was that MSCs enhanced CCL8 expression in these mice.

How did MSCs enhance the expression of CCL8 and inhibit expression of other chemokines including CCL5 in the kidney? To address this issue, we isolated tubular cells and podocytes from the kidney of MRL.*Fas*^lpr^ mice and co-cultured them with MSCs. We also added TNF-α to mimic inflamed conditions. Podocytes and tubular cells produce CCL2, CCL5, CCL7, CCL20, CXCL1, CXCL2, CXCL5, and CXCL16 upon stimulation with lipopolysaccharide, polyI:C, diverse cytokines including TNF-α, autoantibodies, and immune complexes^23,24^. However, there have been no reports on whether MSCs affect chemokine expression by renal cells, although several reports have shown the protective effects of MSCs on podocytes and tubular cells^25–27^. Han et al. reported that MSCs ameliorate podocyte injury by inhibiting PINK1/Parkin-mediated mitophagy in diabetic kidney disease^25^. Wang et al. reported that MSC-derived exosomes containing miR-22-3p protect podocytes from injury and inhibit the production of inflammatory cytokines such as IL-1β and TNF-α by podocytes^26^. Perico et al. reported that MSCs transplanted into mice stimulate tubular cells to regain mitochondrial mass and function^27^. These reports generally show the effects of MSCs on proliferation, apoptosis, and regeneration of renal cells, but not on chemokine expression. In this study, we newly demonstrated that MSCs inhibited CCL5 expression by podocytes and tubular cells, but enhanced CCL8 expression only by podocytes. We also demonstrated the mechanisms of action of MSCs in podocytes: they up-regulated CCL8 expression in a VLA-4-dependent manner and down-regulated CCL5 expression in a TGF-β1-dependent manner.

What are the implications of such effects of MSCs? CCL8 is expected to play a pro-inflammatory role, as it induces the migration of CD4^+^ T cells to the skin in a chronic atopic dermatitis model and the migration of macrophages to the inflamed intestine in a dextran sulfate sodium–induced colitis model^28,29^. However, our data imply that CCL8 might have an anti-inflammatory function and be required for MSCs to efficiently ameliorate nephritis. As a mechanism, we suggest that CCL8 activates MSCs to enhance the production of immunosuppressive soluble factors, such as TGF-β1, IDO, IL-10, and iNOS. CCL5 is produced by activated T cells, renal epithelial cells, fibroblasts, and mesangial cells^30^. It is a potent chemoattractant for T cells and monocytes, which enhances inflammation^30^. CCL5 level is significantly higher in serum of SLE patients than in normal controls^31^. In addition, CCL5 level is significantly associated with those of serum IgG, C3, C4, and anti-dsDNA antibodies in SLE patients^31^. We showed that MSCs inhibited CCL5 expression by podocytes in a TGF-β1-dependent manner, which might ameliorate nephritis by reducing leukocyte migration. Overall, our data suggest that dual regulation of MSCs by up-regulation of CCL8 and down-regulation of CCL5 might work synergistically to ameliorate nephritis.

The implications of our study are limited by several caveats. First, the chemokine network might show complex redundancy^32^. For example, CCL8 can act as a ligand of CCR1, CCR2, CCR3, and CCR5^32^. Thus, it will be interesting to study whether podocyte-derived CCL8 binds all these receptors, which of them are expressed on MSCs or not. Second, we ruled out CCL8 expression by renal-infiltrating immune cells in the kidney of MRL.*Fas*^lpr^ mice. Renal-infiltrating macrophages, dendritic cells, and T cells can produce CCL2, CCL5, CCL8, and CXCL13^21,33^. It will be interesting to study which immune cells in the nephritic kidney express CCL8 and whether its expression is increased by MSCs. Third, the difference between human and mouse makes it difficult to translate the mouse data to clinical trials. For example, although they bind the same CXCR3 receptor, CXCL9 is predominant in lupus-prone mice but CXCL10 is predominant in SLE patients^34^. Therefore, it is important to study the differences and similarities between lupus-prone mice and SLE patients in terms of their use of chemokines and chemokine receptors. Fourth, it will be interesting to investigate the therapeutic effect and mechanisms of action of MSCs in alternative animal models, such as NZB/W F1 mice, and we are planning such experiments to validate the current conclusion derived from lupus-prone MRL.*Fas*^lpr^ mice.

## Conclusion

The results of this study document previously unrecognized mechanisms through which MSCs ameliorate nephritis. MSCs might cooperate with renal cells in the nephritic kidney in two ways. First, MSCs might inhibit CCL5 production by podocytes and tubular cells; CCL5 is a key chemokine inducing the infiltration of inflammatory leukocytes into the kidney. Second, MSCs complementarily cooperate with podocytes: MSCs activate podocytes to produce CCL8, which in turn activates MSCs to increase the production of immunosuppressive factors in a paracrine fashion. Our data imply that CCL5 and CCL8 might be good targets for improving the therapeutic efficacy of MSCs in ameliorating lupus nephritis.

Our data will open new research perspectives. It will be interesting to develop an activator of CCL8 expression in podocytes and its receptors on MSCs, which would potentiate the efficacy of MSCs for the treatment of nephritis. In addition, it will be interesting to develop an inhibitor of CCL5 expression in podocytes and its receptors on MSCs. However, the complex redundancy of the chemokine network might be a major challenge for the development of these kinds of inhibitors^32^. For example, CCL5 can act as a ligand of several receptors (CCR1, CCR3, and CCR5) and CCR5 can interact with different chemokines (CCL3, CCL4, CCL5, and CCL8)^35^. In addition, leukocytes always express more than one chemokine receptor, so blocking the ligation of one receptor may not completely or efficiently prevent leukocyte infiltration. Simultaneous blocking of multiple receptors might be an alternative, but would increase the likelihood of side effects. In addition, chemokines, such as CCL2 and CXCL10, have been suggested as diagnostic biomarkers of lupus nephritis to supplement renal biopsy^36^. It will be interesting to validate the potential use of CCL5 and CCL8 as alternative biomarkers to predict the efficacy of MSCs.

## Materials and methods

### Generation of human and mouse MSCs

Human bone marrow (BM)-derived MSCs were obtained from Corestem Inc. (Gyeonggi, Korea)^14^. In brief, BM cells were aspirated from the posterior iliac crest of healthy human subjects^14^. Mononuclear cells were collected by density gradient centrifugation (Ficoll-Paque; GE Healthcare Bio-Sciences AB, Uppsala, Sweden) and were cultured at 2 × 10^7^ cells/T175 flask in CSBM-A06 medium (Corestem Inc.) containing 10% fetal bovine serum (FBS; Corning, Glendale, AZ, USA), 2.5 mM l-glutamine, and penicillin/streptomycin (WelGene, Gyeonggi, Korea) in a 5% CO_2_ incubator at 37 °C. Medium was changed every 3–4 days and non-adherent cells were removed. Adherent cells were sub-cultured on day 10 or 11 (passage 1). MSCs were used in experiments at passages 3–5. The surface marker profile of MSCs was CD29^+^CD44^+^CD73^+^CD105^+^CD90^+^CD34^−^CD45^−^HLA-DR^−^ (data not shown). All human MSC studies were approved by the Institutional Review Board of Hanyang University Hospital and were carried out in accordance with the approved guidelines. All participants provided written informed consent.

Mouse MSCs were generated from the BM cells of tibiae and femurs of 6–8-week-old C57BL/6 mice (Orient Bio, Gyeonggi, Korea). Red blood cells were lysed with ACK buffer (Thermo Fisher Scientific, Waltham, MA, USA), and BM cells were cultured at 1 × 10^7^ cells/well of a 6-well plate in α-MEM medium (Thermo Fisher Scientific) containing 10% FBS (Corning), 2 mM l-glutamine, and penicillin/streptomycin in a 5% CO_2_ incubator at 37 °C. Medium was changed every 3 days and non-adherent cells were removed. Adherent cells were sub-cultured on day 10 or 11 (passage 1) and used in experiments on day 17–21 (passages 2 or 3)^14^. The surface marker profile of MSCs was Sca-1^+^CD44^+^CD73^+^CD45^−^CD11b^−^CD11c^−^Gr-1^−^MHC-II^−^ (data not shown). All animal studies were approved by the Chungbuk National University Animal Experimentation Ethics Committee and were carried out in accordance with the approved guidelines. All procedures were performed in accordance with ARRIVE guidelines.

### Isolation of podocytes and tubular cells

Podocytes were isolated from kidney of MRL.*Fas*^lpr^ mice at the age of 12 weeks as previously described^37^. Kidney was minced into small pieces and digested with 2 mg/ml of collagenase (Sigma Aldrich, St. Louis, MO, USA) in RPMI-1640 medium (Thermo Fisher Scientific) containing 10% FBS (Corning) at 37 °C for 40 min. After washing with the same medium without collagenase, the specimens were treated with ACK lysis buffer to remove red blood cells. After washing, the specimens were treated again with 0.5 mg/ml of collagenase, 0.5 mg/ml of dispase II, and 0.075% trypsin (all from Sigma Aldrich) in the same medium at 37 °C for 20 min. Single cells were obtained by passing the samples through a 25-μm filter to remove tissue debris. CD31-positive endothelial cells were removed using anti-CD31 antibody-coated MicroBeads (Miltenyi Biotec, Bergisch Gladbach, Germany). CD31-negative cells were labelled with biotin-conjugated anti-nephrin antibody (R&D Systems, Minneapolis, MN, USA), followed by incubation with streptavidin-coated MicroBeads. Nephrin-positive cells were collected using a MACS separation kit (Miltenyi Biotec) and cultured on collagen I–coated dishes.

Tubular cells were isolated from kidney of MRL.*Fas*^lpr^ mice at the age of 12 weeks as previously described^23^. Briefly, digestion buffer was prepared by dissolving 3.9 mg of collagenase (Sigma Aldrich) in 30 ml of PBS and warmed up in a water bath at 37 °C until the start of the isolation procedure. Renal Epithelial Cell Growth Basal Medium 2 (500 ml) supplemented with 0.05 ml FBS (Corning), 10 ng/ml of epidermal growth factor, 5 μg/ml of insulin, 0.5 μg/ml of epinephrine, 36 ng/ml of hydrocortisone, 5 μg/ml of transferrin, and 4 pg/ml of triiodo-l-thyronine (all from PromoCell GmbH, Heidelberg, Germany) was used as culture medium. Mice were perfused with 30 ml of digestion buffer for 1 min. Kidney was then isolated, deprived of renal capsules and medulla, minced into small pieces, and incubated in 10 ml of digestion buffer with gentle rotation for 5 min in a water bath at 37 °C. Undigested tissues were removed by filtering through a 70-μm filter, and the supernatant was mixed with culture medium to stop the digestion. Cell suspension was centrifuged at 50 × *g* for 5 min to collect tubular cells (the first pellet). The supernatant was centrifuged again at 50 × *g* for 5 min to collect remaining tubular cells (the second pellet). The first pellet was washed with 20 ml of medium by centrifugation at 50 × *g* for 5 min (the third pellet). Tubular calls (the second and third pellets) were combined and cultured on dishes pre-coated with collagen^23^.

### Animal experiments

Female MRL*.Fas*^lpr^ (MRL*.*MpJ-*Tnfrsf6*^*Faslpr*^/J) mice were purchased from the Jackson Laboratory (Bar Harbor, ME, USA). Mice were housed in specific pathogen–free conditions at 21–24 °C and 40–60% relative humidity under a 12 h light/dark cycle. In our first experiment (Fig. 1A–D), mice were divided into the following groups: control (PBS, n = 6), MSCs (1 × 10^6^ cells/injection, n = 6), and cyclophosphamide (50 mg/kg, n = 6). Injections were performed intravenously three times at 3-week intervals from the age of 12 weeks. Urine and serum were collected every 2 weeks and stored at − 70 °C until used. The levels of protein in urine and anti-dsDNA IgG and total IgG in serum were measured by using ELISA kits purchased from Sigma-Aldrich, Alpha Diagnostic International (San Antonio, TX, USA), and eBioscience (San Diego, CA, USA), respectively, according to the manufacturers’ instructions. Mice were sacrificed at 20 weeks of age and their spleens were isolated. Expression levels of cytokines in the spleens were determined by RT-PCR^38^.

In the second experiment (Fig. 1E and F), mice were randomly divided into two groups: control (PBS, n = 6) and MSCs (1 × 10^6^ cells/injection, n = 6). Injections were performed intravenously at the age of 12 and 14 weeks. Kidneys were isolated from the injected mice at the age of 16 weeks and also from the control mice at the age of 9 weeks (n = 6). Expression levels of cytokines and chemokines in the kidney were determined by RT-qPCR^14^.

In the third experiment (Fig. 2A), the kidney was isolated from mice at the age of 9 and 18 weeks, fixed with 4% formalin, and immersed in PBS. After dehydration with ethanol and xylene, the tissues were embedded in paraffin and cut into 4-μm sections. After removing paraffin, sections were incubated with the primary antibody against CCL8 (1:500; Bioss, MA, USA) at 4 °C overnight. The sections were then incubated with anti-rabbit IgG antibody conjugated with horseradish peroxidase for 1 h at room temperature (Vector Laboratories, Burlingame, CA, USA)^38^.

In the fourth experiment (Fig. 3C), human MSCs (1 × 10^6^ cells/mouse, n = 6) were injected intravenously into mice at the age of 16 weeks. Kidney was isolated 24 h later, fixed with 4% formalin, and immersed in PBS. After dehydration with ethanol and xylene, the tissues were embedded in paraffin and cut into 4-μm sections. After removing paraffin, kidney sections were incubated with the primary antibodies—rabbit anti-mouse nephrin antibody (1:1,000; Abcam, Cambridge, UK) and mouse anti-human mitochondria antibody (1:250; Abcam)—at 4 °C overnight. Then, all sections were incubated with the secondary antibodies—anti-rabbit IgG conjugated with GFP (green) and anti-mouse IgG conjugated with Alexa Fluor 647 (red)—for 1 h at room temperature. All microscopic images were recorded using a confocal microscope (Zeiss, Oberkochen, Germany)^39^.

### Kidney tissue culture

Kidney was isolated from mice at the age of 9 and 18 weeks. The renal capsule was removed in cold Hanks Balanced Salt Solution (Invitrogen, Carlsbad, CA, USA). The remaining tissues were minced into small pieces and 100-mg pieces were incubated in 5 ml of RPMI-1640 medium with 10% FBS (Corning) in a 6 well-plate at 37 °C for 24 or 48 h. The amount of CCL8 in the medium was measured by ELISA (Bio-Techne, Minneapolis, MN, USA)^40^.

### ELISA, RT-PCR, RT-qPCR, and RNA interference

The levels of CCL5 and CCL8 in culture medium were measured by ELISA (Bio-Techne). The mRNA levels of chemokines, soluble factors, and adhesion molecules were determined by RT-PCR and RT-qPCR. Total RNA was extracted by using TRIZOL Reagent (Thermo Fisher Scientific). cDNA was synthesized from 1 µg RNA using RT PreMix (Bioneer, Daejeon, Korea). PCR was performed as previously described and PCR products were separated on 1% agarose gels and stained with 0.5 mg/ml ethidium bromide^41^. Quantitative PCR was performed with SYBR Green Master Mix (Elpis Biotech, Daejeon, Korea) in a StepOnePlus Real-Time PCR System (Applied Biosystems, Foster City, CA, USA). Determination of the relative mRNA level in a sample was based on its threshold cycle in comparison with that of the housekeeping gene for β-actin^42^. Primer sequences are listed in Supplementary Table 1^43^. MSCs were transfected with 100 nM siRNAs (Bioneer) using Lipofectamine RNAiMAX reagent (Thermo Fisher Scientific) for 48 h at 37℃ in 5% CO_2_. Sequences of siRNAs are listed in Supplementary Table 2^14^.

### Migration assay

T cells, B cells, and NK cells were isolated from the spleen of MRL.*Fas*^lpr^ mice as previously described^13^. Migration of T cells, B cells, and NK cells was examined by using transwell plates with 5-μm pore filters (Corning, Cambridge, MA, USA). Immune cells (1 × 10^5^ cells in 100 μl) were added to the upper wells and CCL5 or CCL8 (600 μl; concentrations are indicated in figure legends) was added to the lower wells. The number of cells that migrated to the lower wells over 2 h was counted using a flow cytometer.

MSC migration was examined by using transwell plates with 8-μm pore filters. Upper wells were pre-coated with 0.1% gelatin (Sigma-Aldrich) for 2 h at 37 °C. MSCs (2 × 10^4^ cells in 100 μl) were added to the upper wells and CCL5 or CCL8 (600 μl; concentrations are indicated in figure legends) was added to the lower wells. After 24-h incubation, non-migrated MSCs on the upper side of the filters were removed by rinsing and the filters were fixed in 10% formalin. MSCs that migrated to the lower side of the filter were stained with 0.5% crystal violet for 10 min and were counted under an inverted light microscope^44^.

### Prevention of cell–cell contacts

In Figs. 3A and 5A, we used transwell plates with 0.5-μm pore filters, which prevented the migration of MSCs in the upper wells to podocytes in the lower wells while allowing the free diffusion of MSC-derived soluble factors^12^.

### Binding assay

TNF-α-treated podocytes were labeled with 0.5 mM CMFDA (Life Technologies, Grand Island, NY, USA) and MSCs with 5 mM CMTPX (Thermo Fisher Scientific) in serum-free medium for 10 min at 37 °C. Labeled cells were washed twice in culture medium containing 10% FBS (Corning). Podocytes (1 × 10^6^ cells) and MSCs (1 × 10^6^ cells) were mixed and centrifuged at 800 rpm for 1 min. The pellets were incubated at 37 °C for 10 min and then gently suspended and analyzed by flow cytometry^44^.

### IFN-γ production by T cells

T cells were purified from spleen cells of MRL*.Fas*^lpr^ mice by a negative depletion method using biotinylated antibodies specific for B220, GR-1, and CD11c (BD Biosciences) and Dynabeads M-280 Streptavidin (Thermo Fisher Scientific). Purity was typically > 90%. Purified T cells (1 × 10^5^ cells/well) were activated with concanavalin A (Con A, 1 μg/ml) or CD3/CD28 antibodies (1 μg/ml each) for 72 h. IFN-γ level in the medium was determined by ELISA (Biotechne)^14^.

### Statistics

Data represent the mean ± SEM of at least three independent experiments, each performed in triplicate. Six mice per group were used in animal study. To determine statistical significance, *p*-values were calculated by one-way ANOVA (GraphPad Prism software, GraphPad Software Inc., San Diego, CA, USA).

### Supplementary Information

Supplementary Tables.

## Data Availability

Datasets for this study are available from the corresponding author upon reasonable request.
