# Supplementary material for: Mesenchymal stem cells enhance CCL8 expression by podocytes in lupus-prone MRL.Faslpr mice
Source: Sci Rep. 2023 Aug 11;13:13074. doi: 10.1038/s41598-023-40346-8 (PMC10421856; doi:10.1038/s41598-023-40346-8)
Supplement: Supplementary file 1 — Supplementary Tables. [file 41598_2023_40346_MOESM1_ESM.docx]

**Supplementary Tables**

Table 1. Primer sequences for RT-PCR and RT-qPCR.

| Molecules Primers |
| --- |
| Chemokines  CCL2 sense, 5′- CCC AAT GAG TAG GCT GGA GA -3′  anti-sense, 5′- GAA CTG CCT TGC CTT CTTG -3′  CCL3 sense, 5′- TCA GCG CCA TGT GAG TCT AC -3′  anti-sense, 5′- GGT TAG ACC CTT CCA CAC CA -3′  CCL4 sense, 5′- CCC ACT TCC TGC TGT TTC TC -3′  anti-sense, 5′- CCC CAT GAC CTC ACT GTT CT -3′  CCL5 sense, 5′- AGA TCT CTG CAG CTG CCC TC -3′  anti-sense, 5′- GGA GCA CTT GCT GCT GGT GT -3′  CCL7 sense, 5′- GCT CAT AGC CGC TGC TTT C -3′  anti-sense, 5′- GCT TTG GAG TTG GGG TTT TC -3′  CCL8 sense, 5′- CCA GAT AAG GCT CCA GTC AC -3′  anti-sense, 5′- AGA GAG ACA TAC CCT GCT TG -3′  CCL11 sense, 5′- TCC ACA GCG CTT CTA TTC CT -3′  anti-sense, 5′- CTA TGG CTT TCA GGG TGC AT -3′  CCL17 sense, 5′- CTG CTC TGC TTC TGG GGA C -3′  anti-sense, 5′- TGT TTG TCT TTG GGG TCT GC -3′  CCL19 sense, 5′- TCT CCT CCC TCC CCT TAG AA -3′  anti-sense, 5′- CGG CTT TAT TGG AAG CTC TG -3′  CCL20 sense, 5′- TGC TCT TCC TTG CTT TGG CA -3′  anti-sense, 5′- TCT GTG CAG TGA TGT GCA GG -3′  CXCL9 sense, 5′- CAG CAA GAT GCC AGA AAA CA -3′  anti-sense, 5′- TGG CTG ATC TGC AAG AAA TG -3′  CXCL10 sense, 5′- GGA TGG CTG TCC TAG CTC TG -3′  anti-sense, 5′- ATA ACC CCT TGG GAA GAT GG -3′  CXCL11 sense, 5′- CAG TCC TCA ATG CCT GTT CA -3′  anti-sense, 5′- GAG CTT GTG GAG CAT TGT CA -3′  CXCL12 sense, 5′- CTT CAT CCC CAT TCT CCT CA -3′  anti-sense, 5′- GAC TCT GCT CTG GTG GAA GG -3′  CX3CL1 sense, 5′- CCG CGT TCT TCC ATT TGT GT -3′  anti-sense, 5′- GCA CAT GAT TTC GCA TTT CG -3′  Cytokines  IL-1β sense, 5′- ATG GCA ATG TTC CTG AAC TC -3′  anti-sense, 5′- CAG GAC AGG TAT AGA TTC TT -3′  IL-2 sense, 5′- CTT GCC CAA GCA GGC CAC AG -3′  anti-sense, 5′- GAG CCT TAT GTG TTG TAA GC -3′  IL-6 sense, 5′- ATC TTT TAC CTC TTG GTT GAA GAT ATG A -3′  anti-sense, 5′- AAA GAG TTG TGC AAT GGC AAT TC -3′  IL-10 sense, 5′- ATA ACT GCA CCC ACT TCC CA -3′  anti-sense, 5′- GGG CAT CAC TTC TAC CAG GT -3′  IFN-γ sense, 5′- AGC GGC TGA CTG AAC TCA GAT TGT AG -3′  anti-sense, 5′- GTC ACA GTT TTC AGC TGT ATA GGG -3′  TNF-α sense, 5′- ACG GCA TGG ATC TCA AAG AC -3′  anti-sense, 5′- GGT CAC TGT CCC AGC ATC TT -3′  Soluble factors  COX-2 sense, 5′- CAG AAC CGC ATT GCC TCT G -3′  anti-sense, 5′- TTG TAA CTT CTG GTC CTC ATG TCG A -3′  IDO sense, 5′- CAC TGA GCA CGG ACG GAC TGA GA -3′  anti-sense, 5′- TCC AAT GCT TTC AGG TCT TGA CGC -3′  iNOS sense, 5′- CCT TCC GAA GTT TCT GGC AGC AGC -3′  anti-sense, 5′- GGC TGT CAG AGC CTC GTG GCT TTG G -3′  HO-1 sense, 5′- CGC AAC AAG CAG AAC CCA -3′  anti-sense, 5′- TGA CGC CAT CTG TGA GGG -3′  TGF-β sense, 5′- AAC AAT TCC TGG CGT TAC CTT -3′  anti-sense, 5′- CTG CCG TAC AAC TCC AGT GA -3′  VEGF sense, 5′- AAC GAA AGC GCA AGA AAT CC -3′  anti-sense, 5′- GCT CAC AGT GAA CGC TCC AG -3′  Adhesion molecules  ICAM-1 sense, 5′- AGA TCA CAT TCA CGG TGC TG -3′  anti-sense, 5′- CTT CAG AGG CAG GAA ACA GG -3′  VCAM-1 sense, 5′- TGG CTC CAG ACA TTT ACC CAG TTT -3′  anti-sense, 5′- GTT CTT TGA CAG TCT CCC TTT CTT T -3′  CD80 sense, 5′- CTT TGG CTA TGG GCT TCC AGT C -3′  anti-sense, 5′- GCA AGG AGG ACA GAG TTT ATC GTG -3′  LFA-1 sense, 5′- TCC TTC CGG AAA GTG GAG AT -3′  anti-sense, 5′- GAG CTC CTC GCA GCT CAC -3′  Mac-1 sense, 5′- ATG GAC GCT GAT GGC AAT ACC -3′  anti-sense, 5′- TCC CCA TTC ACG TCT CCC A -3′  VLA-4 sense, 5′- CAC TCC AGC CGA TCC TTC A -3′  anti-sense, 5′- TGC AGG CAA GCT TCA CTA TG -3′  CD28 sense, 5′- CAG TCG CCC TGC TTG TGG TAG ATA GC -3′  anti-sense, 5′- AGT TCC ATT GCT CCT CTC GTT GTC -3′  β-actin sense, 5′- TGG AAT CCT GTG GCA TCC ATG AAA C -3′  anti-sense, 5′- TAA AAC GCA GCT CAG TAA CAG TCC G -3′ |

Table 2. siRNA sequences.

| Molecules Primers |
| --- |
| IL-10 Seq 1, 5′- GAC UCC UUA AUG CAG GAC U -3′  Seq 2, 5′- GAG ACU UGC UCU UGC ACU A -3′  Seq 3, 5′- GAC CUA GAC AGA GCU CUC U -3′  IDO Seq 1, 5′- CUG AGU UGG CCU UAG UGU A -3′  Seq 2, 5′- CUG UAU CCA GUG CAG UAG A -3′  Seq 3, 5′- UCU GUC UUA UGC AGA UCG U -3′  TGF-β Seq 1, 5′- GUG GAA AUC AAC GGG AUC A -3′  Seq 2, 5′- CUC ACU GCU CUU GUG ACA G -3′  Seq 3, 5′- CGG UGC UCG CUU UGU ACA A -3′  HO−1 Seq 1, 5′- AAG GAC AUG GCC UUC UGG U -3′  Seq 2, 5′- AAU GAA CAC UCU GGA GAU G -3′  Seq 3, 5′- AAG ACC AGA GUC CCU CAC A -3′  iNOS Seq 1, 5′- CAG AGU ACA AAG ACA CGA U -3′  Seq 2, 5′- GAC CAA GAA GUC AUU GCU U -3′  Seq 3, 5′- GUG GUU UUU AGG UAG AUG U -3′  IL-6 Seq 1, 5′- CAG AAA CCG CUA UGA AGU U -3′  Seq 2, 5′- GAG GAU ACC ACU CCC AAC A -3′  Seq 3, 5′- GAG UUG UGC AAU GGC AAU U -3′  Nagative Seq 1, 5′- CCU ACG CCA CCA AUU UCG U(dTdT) -3′ |
